# Molnupiravir When Used Alone Seems to Be Safe and Effective as Outpatient COVID-19 Therapy for Hemodialyzed Patients and Kidney Transplant Recipients

**DOI:** 10.3390/v14102224

**Published:** 2022-10-09

**Authors:** Paweł Poznański, Hanna Augustyniak-Bartosik, Anna Magiera-Żak, Karolina Skalec, Katarzyna Jakuszko, Oktawia Mazanowska, Dariusz Janczak, Magdalena Krajewska, Dorota Kamińska

**Affiliations:** 1 Department of Nephrology and Transplantation Medicine, Wroclaw Medical University, Borowska 213, 50-556 Wrocław, Poland; 2 Department of Vascular, General and Transplant Surgery, Wroclaw Medical University, Borowska 213, 50-556 Wrocław, Poland

**Keywords:** COVID-19, SARS-CoV-2, molnupiravir, ESKD, ESRD, kidney transplantation, hemodialysis

## Abstract

Background: Molnupiravir demonstrated an in vitro antiviral activity against positive-sense RNA viruses, including SARS-CoV-2. The study aimed to present the results of outpatient molnupiravir use in kidney transplant recipients and hemodialysis patients during the first months of 2022 in Poland. Methods: The retrospective observational cohort study at one kidney transplant center included 36 patients diagnosed with COVID-19 with an automated nucleic acid amplification test on nasopharyngeal swab specimens. All patients received molnupiravir for home-based therapy at a dose of 800 mg every 12 h orally for 5 days. Both kidney transplant recipients (*n* = 16) and hemodialysis patients (*n* = 20) presented a lot of comorbidities with a Charlson comorbidity index of 4.1 and 5.1, respectively. Results: Patients presented with fever, cough, and weakness followed by muscle and joint pain. Five kidney transplant recipients experienced acute kidney injury with a rise in serum creatinine level from 0.4 to 1.9 mg/dL. No serious side effects of molnupiravir therapy or interactions with immunosuppressive medications were observed. Symptoms of COVID-19 improved rapidly or resolved within 24–48 h of starting treatment. Conclusion: The study suggests the safety and efficacy of molnupiravir therapy alone early after the onset of SARS-CoV-2 infection, but further investigations should be performed to confirm our preliminary results. To the best of the authors’ knowledge, it is the first published report on molnupiravir use in end-stage kidney disease (ESKD) patients on hemodialysis and the third concerning kidney transplant recipients.

## 1. Introduction

The SARS-CoV-2 pandemic is a serious threat, especially in vulnerable populations, including immunocompromised patients. Patients with end-stage kidney disease (ESKD) treated with hemodialysis (HD), and kidney transplant recipients (KTRs) are at high risk of COVID-19-related complications and mortality [1,2]. At the beginning of the COVID-19 pandemic, the European Renal Association Registry reported mortality of 20.0% for patients on dialysis [3]. The COVID-19 mortality rate among kidney transplant recipients exceeded levels reported for the general population and patients on HD and was reported to range from 17 to even 28% [4]. 

Also, the response to vaccination in HD patients and KTRs is much lower than in the general population [5]. For the aforementioned reasons, using effective drugs against COVID-19 in these patient groups is essential. New drugs introduced during the COVID-19 pandemic were also used in KTR and HD patients; however, some presented serious adverse effects and interactions with immunosuppressive medication. KTRs are an incoherent group of patients when considering graft function, underlying condition, and immunosuppressive regimen. Moreover, deteriorated kidney function can affect the tolerability and efficacy of antiCOIVD-19 medications. Initially, it was stated in the manufacturer’s labeling that remdesivir was not recommended for severe kidney impairment [6]. Similarly, the baricitinib dose adjustment is required for impaired kidney function, and drug–drug interactions should be considered during administration in KTRs. The real-life experience with oral antiviral drugs in these groups of patients is being analyzed. 

Molnupiravir exerts antiviral activity by inhibiting RNA-dependent RNA polymerase, and it has demonstrated an in vitro antiviral activity against positive-sense RNA viruses, including SARS-CoV-2 [7]. During the COVID-19 pandemic, molnupiravir demonstrated good efficacy as well as a good safety profile. Molnupiravir reduced the risk of hospitalization or death by 30% in patients with COVID-19 and has been authorized for emergency use by the UK Medicines and Healthcare products Regulatory Agency (MHRA) and the US Food and Drug Administration (FDA) in adults with mild to moderate COVID-19 [8]. No dose adjustment of molnupiravir is required in cases of mild chronic kidney disease; however, the patients with severe kidney failure were not included in the clinical trials [9]. Two studies have reported good clinical efficacy of molnupiravir with no serious side effects among transplant recipients [10,11]. No study of molnupiravir use among hemodialysis patients has been published.

This paper presents the results of outpatient molnupiravir use in KTRs and HD patients during the first months of 2022 in Poland. 

## 2. Materials and Methods

A retrospective observational cohort study was performed at one kidney transplant center with an outpatient clinic for 1200 adult kidney transplant recipients. Under a regulation of the Polish Ministry [9] of Health, since 15 January 2022, all adult KTRs and HD patients diagnosed with SARS-CoV-2 infection with automated nucleic acid amplification test on nasopharyngeal swab specimens (NPS) were considered for molnupiravir therapy. The local government advisory boards recommended treatment initiation in patients with proven SARS-CoV-2 infection who do not require oxygen therapy and belong to the risk groups.

All patients who contacted the transplant/dialysis center between 1 February 2022 and 30 April 2022 were eligible for the treatment and provided informed written consent. 

Inclusion criteria according to the therapeutic indication authorized by the Polish Ministry of Health concerned all the patients at risk of progression to severe COVID-19, including KTRs and HD patients older than 18 years and diagnosed with SARS-CoV-2 infection within the first 5 days, with mild to moderate symptoms, which could be treated as outpatients. Exclusion criteria included the severity of symptoms requiring hospitalization, and impaired kidney allograft function was not an exclusion criterion. 

Out of 40 patients who received molnupiravir, four were excluded from the study due to insufficient data. The clinical characteristics of 36 patients with medical history, laboratory data, treatments received, kidney allograft function, and clinical resolution of SARS-CoV-2 infection was included in the study database. The study followed the principles of the Declaration of Helsinki, formulated by the World Medical Association.

All the patients received molnupiravir for home-based therapy at a dose of 800 mg every 12 h orally for 5 days. None of the patients refused the therapy.

After the centralized distribution of the drug was discontinued, clinical and laboratory data were collected. The questionnaire regarding the course of COVID-19, symptoms of the disease, and side effects of Molnupiravir therapy was conducted by phone or during a visit to the center within three months after completion of the therapy (Table S1: Molnupiravir questionnaire).

## 3. Results

According to the epidemiological data, the most prevalent SARS-CoV-2 variant in Poland in Poland during the time of the study was the B.1.1.529 (Omicron) variant [12,13]. Patients were not tested for the evaluation of the SARS-CoV-2 variant.

The sample group included 16 KTRs (12 females, 4 males with a mean age of 49 y.) and 20 HD patients (7 females, 13 males with a mean age of 58 y.). KTRs presented satisfying kidney allograft function before SARS-CoV-2 infection (with a mean serum creatinine level of 1.6 mg/dL). They were from 1 month to 22 years after transplantation (the mean time from transplant to the infection with SARS-CoV-2 diagnosis was 86 months). Both KTRs and HD patients presented a lot of comorbidities with a Charlson comorbidity index of 4.1 and 5.1, respectively. Characteristics of recipients treated with molnupiravir are presented in Table 1. 

### 3.1. COVID-19 Symptoms

Twelve patients did not develop clinical symptoms of COVID-19 and were diagnosed during routine diagnostics performed in the hospital or an outpatient clinic. None of the patients presented anosmia, ageusia, respiratory failure, or diarrhea. Most commonly, they reported fever, cough, and weakness followed by muscle and joint pain. Five KTRs (20%) experienced AKI at the time of diagnosis, with a rise in creatinine serum level from 0.4 to 1.9 mg/dL. All the recipients recovered and returned to the previous kidney allograft function. However, onset of sub-nephrotic proteinuria was observed in the first months after the SARS-CoV-2 infection in two patients. 

### 3.2. Relation to Previous Anti-SARS-CoV-2 Vaccination

A total of 33 patients were previously vaccinated with the mRNA anti-SARS-CoV-2 vaccine, where most had three doses (69% of KTRs, 50% of HD patients). Three patients (two KTRs, and one HD patient) refused to be vaccinated in the past. The mean time from the last vaccination to infection was 4 months for KTRs and 6 months for HD patients. 

### 3.3. Modification of Immunosuppression

After the diagnosis, in all the KTRs, mycophenolate mofetil/sodium was temporarily suspended (in nine cases), or the dose was reduced (in four cases). In most KTRs (69%), an increase in glucocorticoid dose was recommended (usually from 5 to 10 mg per day per dose of prednisone) [14]. Calcineurin inhibitors (CNI) and mechanistic target for rapamycin inhibitors (mTORi) dosages were not modified during the antiviral treatment period. 

### 3.4. Adherence to Molnupiravir Therapy

The vast majority (over 90%) of the patients fully adhered to the prescribed dose of molnupiravir. Three patients did not complete the full therapy (one patient forgot about the last dose, and two patients used a reduced dose due to fear of drug toxicity). The therapy was not terminated prematurely in any case of patients requiring hospitalization due to COVID-19.

### 3.5. Side-Effects of Molnupiravir Therapy

All the patients were asked about the side effects of molnupiravir, particularly: diarrhea, nausea, vomiting, headaches, dizziness, and rash. All but one patient denied any side effects of molnupiravir. One patient reported mild and transient headaches and dizziness. 

No cases of kidney allograft function deterioration due to molnupiravir therapy were found. The CNI trough concentrations were assessed before the initiation of the molnupiravir therapy and after its completion. No impact on trough levels of CNIs resulting in the necessity of dose modification medications was observed. The pre- and post-treatment median Tacrolimus trough concentration was 6.45 ng/mL (IQR 5.3–7.3, min-max 4.1–13.3) and 6.9 ng/mL (IQR 5,6–7.7, min-max 4.0–11.5), respectively (*p* = 0.625). The observed pre- and post-treatment Cyclosporine A trough concentrations were 124 ng/mL and 115 ng/mL versus 152 ng/mL and 130 ng/mL, respectively. All pre- and post-treatment trough CNIs levels were within patients’ target values. Also, no significant side effects were shown in maintaining hemodialysis during molnupiravir therapy in the sample group.

### 3.6. Efficacy of Molnupiravir Therapy

The most severe symptoms of COVID-19 (fever, muscle and joint pain, severe headache) improved rapidly or resolved within 24–48 h of starting treatment with molnupiravir in all the patients from the group not requiring admission to the hospital due to COVID-19 (i.e., 91.5% in KTRs, and 85% in HD patients). However, some patients had dry cough and weakness for 2–3 weeks. The duration of symptoms in symptomatic patients was twice longer in the HD group compared to KTRs. 

A total of 12.5% of KTRs (2 recipients) required hospitalization due to COVID-19. However, oxygen therapy was required for only one person (6.25%). The reasons for hospitalization in the KTRs group were: fever exceeding 39 °C, dyspnea, or acute kidney allograft injury. Need for hospitalization was observed in 15% of HD patients (three patients), with oxygen therapy in 10%. All the hospitalized HD patients had fever and dyspnea, and one also had hypotension and impaired consciousness. None of the patients died due to COVID-19 in both groups. 

Data concerning the safety and tolerability of molnupiravir are presented in Table 2.

## 4. Discussion

The presented study concerned the safety and efficacy of molnupiravir use in patients with ESKD. It showed that molnupiravir therapy alone early after the onset of SARS-CoV-2 infection is safe and effective. To the authors’ best knowledge, it is the first published report on molnupiravir use in ESKD patients on hemodialysis and the third concerning kidney transplant recipients.

The transmissibility of the last two SARS-CoV-2 variants of concern—B.1.617.2 (Delta) and B.1.1.529 Omicron—are higher than previous lineages. The Omicron variant, first discovered in South Africa, became prevalent in most countries in early 2022 [12]. It was well-established that the Omicron variant has higher transmissibility and infectivity than the Delta variant. The Omicron variant of SARS-CoV-2 is associated with a decrease in mortality rate compared to the previous variants, including the Delta variant. Also, the risk of severe pneumonia is lower than reported during 2020 and 2021 [15]. However, in most countries, daily hospitalization rates due to the Omicron variant during the peak outbreak were higher than in the case of the Delta variant but, at the same time, had less effect on the daily number of ICU cases [16].

The Omicron variant prevailed in Poland in the first 4 months of 2022, with the mortality rate reaching approx. 30 per million population [12], with a lower probability of respiratory failure. However, it was still dangerous for both KTRs and HD patients. Terminal kidney failure, immunosuppressive therapy, and concomitant diseases make the population of HD/KTRs especially vulnerable to severe COVID-19. HD patients develop an impaired cellular and humoral response to COVID-19 vaccination compared to the general population [5]. KTRs show a preserved symptom-dependent humoral response to SARS-CoV-2 infection [17]. However, SARS-CoV-2-reactive T cell response is weaker in KTRs compared to HD patients as well as the general population [18]. Vaccination strategies were also less effective in KTRs and HD patients than in the healthy population [19]. For this reason, using an efficient drug that potentially decreases mortality and death rate is crucial among end-stage kidney disease patients. 

A recent meta-analysis showed that three novel oral antivirals (molnupiravir, fluvoxamine, and ritonavir-boosted nirmatrelvir) effectively reduce mortality and hospitalization rates in patients with COVID-19 [20]. Therapy with molnupiravir initiated early after the onset of SARS-CoV-2 infection was shown to reduce the risk of hospitalization or death in at-risk, unvaccinated adults with COVID-19 [21] as well as in vaccinated vulnerable populations [22]. Molnupiravir was approved for adults with mild to moderate COVID-19 and was shown to reduce the risk of hospitalization or death by 30% [8]. It was reported that the SARS-CoV-2 Omicron variant is highly sensitive to molnupiravir [23,24]. It was also shown that for non-hospitalized patients with COVID-19 who are at risk of disease progression, oral nirmatrelvir plus ritonavir (which was not distributed in Poland at the time of the study) and intravenous remdesivir (which at that time was reserved for patients with respiratory failure) seem to be a better choice, followed by molnupiravir [25]. 

Two papers have been published concerning molnupiravir use in the transplant setting. Radcliffe et al. described 74 solid organ transplant recipients treated with molnupiravir, sotrovimab, or nirmatrelvir/ritonavir. They found that in the molnupiravir-treated group, the hospitalization rate was lower (16%) compared to the control group of 48 transplant recipients with no therapy applied (27%). There were no deaths in those who received any therapy versus three (6%) deaths in patients without outpatient therapy [11]. The hospitalization rate among KTRs in the study presented in this paper was similar. Still, it could not be compared to untreated recipients because, according to the Polish Ministry of Health regulations, all the recipients diagnosed with COVID-19 were scheduled for molnupiravir therapy at that time (Omicron surge). The results obtained by the authors could not be compared with those prior to molnupiravir use due to different SARS-CoV-2 variants at different times, which presented various mortality rates. 

A recent paper by Villamarín et al. compared data of molnupiravir treatment of 9 KTRs with mild COVID-19 and eight KTRs treated with remdesivir. Recipients in both groups presented an excellent clinical evolution with a lower risk for hospitalization and no adverse effects. No evidence of nephrotoxicity secondary to the drug nor interactions with the immunosuppressive therapy were found [10]. This is in line with the observation of molnupiravir safety and efficacy presented in this paper in which, additionally, no worsening of kidney allograft function nor interactions with immunosuppressive medications were noted. The sample group could not be compared with patients treated with remdesivir because treatment with remdesivir in Poland is limited to cases with respiratory failure, and the use of molnupiravir is limited only to mild-to-moderate cases without respiratory failure. 

In this study, results could not be compared with other studies on the use of molnupiravir in hemodialysis patients as no published data on this could be found.

This study has a few limitations. First, this is a retrospective single-center study in a relatively small sample group with no control group due to the reasons described above. However, it describes the real-life experience of molnupiravir use in a setting of ESKD patients showing safety, good tolerability, and efficacy of molnupiravir therapy in that group of patients. 

## 5. Conclusions

The study suggests that molnupiravir therapy alone applied early after the onset of SARS-CoV-2 infection can be safe and well tolerated in the cohort of ESKD patients treated with hemodialysis or kidney transplantation. As the COVID-19 pandemic is not expected to finish in the near future, continued studies of new outpatient therapies are needed, especially in high-risk populations, including ESKD patients.

## Figures and Tables

**Table 1:** Clinical characteristics of sample groups.

Patients Characteristics	KTRs (16)	HD Patients (20)
Age (years, mean ± SD)	49.2 ± 18.95	57.6 ± 19.63
Gender (F/M)	12/4	7/13
BMI (kg/m^2^, mean ± SD)	21.96 ± 3.68	25.79 ± 3.57
Duration of HD therapy (months)	-	19 ± 16
Time since kidney transplantation (months)	86 ± 85	-
Living/deceased donor	2/14	-
**Current immunosuppression** (No. of recipients)		
*Steroids*	16	-
*Tacrolimus/Cyclosporine A*	14/2	-
*MPA/Azathioprine/mTORi/none*	13/1/1/1	-
**Charlson comorbidity index** (mean ± SD)	4.1 ± 1.67	5.1 ± 2.75
**Graft function prior to COVID-19**		
*Serum creatinine* (mg/dL, mean ± SD)	1.64 ± 0.68	-
**Previous vaccination against COVID-19**		
Yes/No	15/1	18/2
1 dose/2 doses/3 doses/4 doses/no data	1/3/11/0/0	2/5/10/0/1
Time from the last vaccination to infection (weeks, ± SD)	23 ± 12	24 ± 13
**COVID-19 symptoms** (% of symptomatic patients)		
Fever	67	33
Cough	56	67
Weakness	22	47
Dyspnoea	22	33
Muscle and joint pain	22	33
Headache	22	20
**No clinical symptoms (% of all)**	44	25
**The duration of the symptoms (days, mean ± SD** **)**	6.6 ± 3.4	12.2 ± 6.8

BMI—body mass index, F—female, HD—hemodialysis, KTRs—kidney transplant recipients M—male, mTORi—mechanistic target for rapamycin inhibitors, MPA—mycophenolic acid,.

**Table 2:** Data concerning safety and tolerability of molnupiravir use in ESKD.

	KTRs	HD Patients
Adherence to the therapy * (%)	94	90
Side effects (%)	0	5 (headaches and dizziness)
Need of hospitalization due to COVID-19 (%)	12.5	15
Need for oxygen therapy (%)	6.25	10
Death (%)	0	0
Acute kidney allograft injury (%)	20	NA

* percentage of the patients who fully adhered to molnupiravir therapy.

## Data Availability

Not applicable.

## Supplementary Materials

The following supporting information can be downloaded at: https://www.mdpi.com/article/10.3390/v14102224/s1, Table S1: Molnupiravir questionnaire.
