# Supplementary material for: Molnupiravir When Used Alone Seems to Be Safe and Effective as Outpatient COVID-19 Therapy for Hemodialyzed Patients and Kidney Transplant Recipients
Source: Viruses. 2022 Oct 9;14(10):2224. doi: 10.3390/v14102224 (PMC9610487; doi:10.3390/v14102224)
Supplement: Supplementary file 1 [file viruses-14-02224-s001.zip › viruses-1900389-Table S1.pdf]

**DATE**	……………………………………..
**NAME**	……………………………………..
**GENDER**	❑ Female ❑ Male
**AGE**	……………………………………..
**BODY MASS**	……………………………………..
**HEIGHT**	……………………………………..
**ESRD underlaying condition**	❑ Diabetic kidney disease<br>❑ Hypertensive nephropathy<br>❑ Glomerulonephritis<br>❑ Cystic/hereditary<br>❑ Unknown<br>❑ Other: ……………………………
**Renal replacement therapy:**	❑ Hemodialysis<br>Start date: ……………………..
**Concomitant diseases:**	❑ Myocardial infarction<br>❑ Congestive heart failure<br>❑ Peripheral vascular disease<br>❑ Cerebrovascular accident<br>❑ Transient ischemic attack<br>❑ Dementia<br>❑ Chronic obstructive pulmonary disease<br>❑ Connective tissue disease<br>❑ Peptic ulcer disease<br>❑ Diabetes mellitus<br>❑ uncomplicated<br>❑ end-organ damage<br>❑ Hemiplegia<br>❑ Chronic kidney disease<br>❑ Liver disease<br>❑ mild = chronic hepatitis (or cirrhosis without portal hypertension)<br>❑ moderate = cirrhosis and portal hypertension but no variceal bleeding history<br>❑ severe = cirrhosis and portal hypertension with variceal bleeding history<br>❑ Solid tumor<br>❑ localized<br>❑ metastatic<br>❑ Leukemia<br>❑ Lymphoma<br>❑ AIDS<br>❑ Other: ……………………………
**Kidney transplantation**	Date: ……………………………………..<br>Donor ❑ Living donor<br>❑ Deceased donor<br>Serum creatinine prior to COVID-19: ……………………
**Immunosuppressive regimen prior to COVID-19:**	❑ Steroids<br>❑ Tacrolimus<br>❑ Cyclosporine A<br>❑ MMF/MPA<br>❑ Azathioprine<br>❑ Sirolimus<br>❑ Everolimus
**Immunosuppressive regimen modification after onset of SARS-CoV-2 infection:**	❑ Dose reduction<br>Drug name: ……………………………………..<br>Dose prior to reduction…………………..<br>Dose after reduction………………………..<br>❑ Drug withdrawal<br>Drug name: ……………………………………..<br>❑ Other: ……………………………………..

**SARS-CoV-2 vaccinations (brand name, dates):**	❑ YES<br>❑ 1 dose (date) ………… brand name ……………<br>❑ 2 doses (date) ……….. brand name ………….<br>❑ 3 doses (date) ……….. brand name……………<br>❑ 4 doses (date) ……….. brand name ……………	❑ NO
**SARS-CoV-2 vaccinations (side effects):**	❑ Pain at the injection site<br>dose number(s):………………<br>❑ Fatigue<br>dose number(s):………………<br>❑ Headache<br>dose number(s):………………<br>❑ Muscle aches<br>dose number(s):………………<br>❑ Arthralgia<br>dose number(s):………………<br>❑ Chills<br>dose number(s):………………	❑ Fever<br>dose number(s):………………<br>❑ Swelling at the injection site<br>dose number(s):………………<br>❑ Redness at the injection site<br>dose number(s):………………<br>❑ Nausea<br>dose number(s):………………<br>❑ Enlarged lymph nodes<br>dose number(s):………………<br>❑ Feeling unwell<br>dose number(s):………………
**COVID-19:**	date of the symptoms onset: ……………………………………<br>symptoms duration (days): ……………………………………<br>date of the positive test: ……………………………………<br>hospitalization due to COVID-19 ❑ YES ❑ NO<br>oxygen therapy ❑ YES ❑ NO<br>minimal oxygen saturation (%)…………………………………	
**COVID-19: symptoms**	❑ anosmia<br>❑ dyspnea<br>❑ fever<br>❑ cough<br>❑ muscle and join pain<br>❑ weakness<br>❑ diarrhea<br>❑ dizziness<br>❑ skin hyperalgesia<br>❑ headaches<br>❑ memory impairment<br>❑ concentration problems	
**MOLNUPIRAVIR THERAPY:**	date of distribution: ……………………………………<br>missed doses ❑ YES ❑ NO<br>if yes, why ……………………………………………………<br>premature therapy discontinuation:<br>❑ WHEN: …………………….. ❑ WHY: ……………………..	
**MOLNUPIRAVIR THERAPY - side effects:**	❑ diarrhea<br>❑ nausea<br>❑ vomiting<br>❑ dizziness	❑ headaches<br>❑ rash<br>❑ other (please describe):<br>……………………………………
